# Inpatient burden of lung cancer and changes after a hospital performance reform: a real-world study

**DOI:** 10.3389/fonc.2025.1771441

**Published:** 2026-01-26

**Authors:** Binbin Han, Xiaofang Chen

**Affiliations:** 1 School of Management, Wuhan University of Technology, Wuhan, Hubei, China; 2 The Affiliated Cancer Hospital of Zhengzhou University & Henan Cancer Hospital, Zhengzhou, Henan, China

**Keywords:** hospital costs, inpatient burden, length of stay, lung cancer, performance reform

## Abstract

**Background:**

Lung cancer places a substantial burden on hospital inpatient care, particularly in tertiary cancer centers. Evidence remains limited on how hospital performance-based management reforms are associated with inpatient efficiency and costs among patients with lung cancer.

**Methods:**

We conducted a retrospective, real-world study using inpatient administrative data from a tertiary cancer hospital in China between 2016 and 2020. Hospitalizations (admissions) of patients with lung cancer were identified, and patient records were linked to enable secondary patient-level analyses. Length of stay (LOS) and daily hospitalization costs were evaluated as complementary indicators of inpatient efficiency and resource utilization intensity. A hospital performance reform implemented in April 2018 was examined by comparing pre-reform (2016–2017) and post-reform (2019–2020) periods. An interrupted time series analysis (ITSA) was conducted using segmented regression on monthly geometric means of log-transformed outcomes at the hospitalization level. Multivariable patient-level regression analyses were conducted as secondary analyses.

**Results:**

A total of 25,331 patients hospitalized with lung cancer were included. After April 2018, LOS declined by approximately 1.6% per month (p < 0.001) relative to the pre-reform trend, while daily hospitalization costs increased by approximately 2.1% per month (p < 0.001) relative to the pre-reform trend. Patient-level analyses were directionally consistent, with the post-reform period associated with a 16.0% shorter LOS and a 31.9% higher daily cost. Sensitivity analyses excluding 2020 and restricting to index admissions yielded similar results.

**Conclusions:**

Among patients hospitalized with lung cancer, the hospital performance reform implemented in 2018 was associated with shorter hospitalization duration and higher daily costs. These findings suggest concurrent changes in inpatient efficiency and resource utilization intensity and highlight the importance of using complementary indicators when evaluating hospital management reforms in oncology care.

## Introduction

Lung cancer remains the leading cause of cancer-related mortality in Asia, accounting for a substantial share of inpatient admissions and healthcare expenditures in tertiary hospitals (1). Despite advances in early detection and treatment, a large proportion of patients continue to present with advanced disease, requiring intensive inpatient care and repeated hospitalizations (2). As a result, lung cancer imposes a considerable burden not only on patients and families but also on hospital systems operating under increasing financial and capacity constraints (3).

In many Asian healthcare settings, tertiary hospitals play a central role in cancer care delivery, concentrating specialized diagnostic and therapeutic resources. Within this context, inpatient length of stay and hospitalization costs have emerged as key indicators for assessing hospital operational performance and resource utilization (4, 5). Length of stay reflects the efficiency of inpatient management, including clinical pathway coordination and bed turnover, whereas total and daily hospitalization costs capture the financial burden and intensity of resource use during routine care (6, 7). Understanding temporal patterns in these indicators is essential for evaluating how hospitals respond to growing cancer-related demands under constrained resources.

In parallel with rising cancer burden, healthcare systems across Asia have increasingly adopted performance-based management reforms to improve efficiency and cost containment (8). In our hospital, a performance reform implemented in 2018 aimed to realign provider incentives by linking remuneration and departmental evaluation to indicators of service efficiency, cost control, and quality of care. Although such reforms are expected to influence hospital operations, empirical evidence on how they are associated with inpatient efficiency and expenditure patterns in oncology care remains limited. Existing studies have primarily focused on aggregate hospital financial performance or outpatient services, with fewer analyses examining disease-specific inpatient outcomes using real-world data (9–11).

Evaluating the impact of management reforms in oncology care poses several challenges. Randomized or quasi-experimental designs are often infeasible in hospital-wide policy changes, and administrative data are subject to heterogeneity in case mix, admission frequency, and clinical severity. Nevertheless, longitudinal hospital records provide a valuable opportunity to examine changes in inpatient utilization and cost patterns before and after reform implementation, offering insights into how performance-based policies may reshape resource allocation in routine cancer care (12).

Using real-world inpatient data from a tertiary hospital, this study assessed the economic burden of lung cancer hospitalizations by examining patterns in length of stay and hospitalization costs. In addition, we evaluated the association between a hospital performance reform implemented in 2018 and changes in these inpatient indicators. This analysis aimed to provide disease-specific evidence on hospital efficiency and resource utilization in routine oncology care.

## Methods

### Study design and data source

This was a retrospective, single-center observational study conducted at a tertiary cancer hospital in China. We used inpatient administrative discharge data containing standardized information on patient demographics, diagnoses, procedures, and hospitalization costs. The study period covered January 2016 to December 2020. This study was reported in accordance with the RECORD statement for studies using routinely collected health data (13).

### Study population

Lung cancer–related hospitalizations were identified using the International Classification of Diseases, 10th Revision (ICD-10) code C34 recorded as either a primary or secondary diagnosis. All eligible inpatient records during the study period were initially identified at the admission level. Records with missing or implausible key information, including admission or discharge dates, length of stay ≤0, or non-positive hospitalization costs, were excluded. A flow diagram summarizing cohort identification, exclusions, and final analytic samples is provided in **Supplementary Figure S1**. For analyses conducted at the patient level, multiple hospitalizations for the same patient were linked using a unique patient identifier.

### Performance reform exposure

The exposure of interest was a hospital-wide performance reform implemented in April 2018. The reform modified the internal performance evaluation and income distribution system, increasing the weight assigned to performance-based components, particularly those related to clinical workload and advanced surgical procedures. For time-series analyses, April 2018 was specified *a priori* as the intervention breakpoint. For descriptive and secondary patient-level analyses using simple pre/post comparisons, hospitalizations were grouped into pre-reform (2016–2017) and post-reform (2019–2020) periods. Data from 2018 were treated as a transition period and were not included in simple pre/post regression models.

### Outcome measures

The primary outcomes were length of stay (LOS) and daily hospitalization cost (14), selected as complementary indicators of inpatient efficiency and resource use intensity. Daily cost was calculated as total hospitalization cost divided by LOS. Total hospitalization cost was analyzed as a secondary outcome and is presented in the **Supplementary Materials**, given its mechanical relationship with LOS and daily cost. All cost variables were adjusted for inflation using the consumer price index, with 2021 as the reference year, and expressed in constant prices (15). Due to right-skewed distributions, LOS and cost outcomes were log-transformed for regression analyses.

### Covariates

Covariates included age at index admission, sex, insurance type, surgical grade, and the number of hospital admissions during the observation window. Insurance type was categorized as Urban Employee Basic Medical Insurance (UEBMI), Urban–Rural Resident Basic Medical Insurance (URRBMI), self-pay, or other payment types. Surgical grade was grouped into low-grade (grades 1–2) and high-grade (grades 3–4) procedures, with no surgery as the reference category. Insurance type and surgical characteristics were defined based on the index admission (first observed hospitalization).

### Statistical analysis

Descriptive analyses were used to summarize patient characteristics and inpatient utilization across study years. The primary evaluation approach was an ITSA using segmented regression models applied to monthly aggregated outcomes. Models estimated both immediate level changes and changes in post-intervention trends (slope changes) associated with the April 2018 reform. Month indicators were included to account for seasonality. As a sensitivity analysis, ITSA was repeated using data restricted to index admissions only (first hospitalization per patient) to assess whether findings were influenced by repeated admissions or differential weighting of frequent utilizers. Secondary analyses included patient-level regression models comparing pre- and post-reform cohorts, adjusting for demographic and clinical covariates and admission frequency, to assess consistency with ITSA findings. Log-transformation was used to address skewed outcome distributions; no additional trimming or winsorization was applied. A two-sided p-value <0.05 was considered statistically significant. All analyses were conducted using R software.

### Ethics statement

This study was approved by the institutional ethics committee of the study hospital. As the analysis was based on retrospective, de-identified administrative data, the requirement for informed consent was waived.

## Results

### Patient characteristics and inpatient burden

A total of 25,331 patients hospitalized with lung cancer were included in the analysis (**Table 1**). The mean age was 60.98 years (SD 10.44), and 64.6% of patients were male. The median number of admissions per patient was 2 (IQR, 1–6). The median total length of stay was 29 days (IQR, 14–61). The median CPI-adjusted total hospitalization cost was 63,575.49, with a median daily cost of 1,747.00. Most patients were covered by urban or rural resident basic medical insurance, while 21.2% were self-paying. Surgical treatment was recorded in 21.1% of patients, and high-grade surgeries accounted for 17.0% of patients.

**Table 1:** Baseline characteristics and inpatient burden among patients hospitalized with lung cancer.

Variables	Overall (*N* = 25331)
Age, mean (SD)	60.98 (10.44)
Sex, % male	16361 (64.6)
Hospitalization characteristics	
Number of admissions, median [IQR]	2.00 [1.00, 6.00]
Total length of stay (days), median [IQR]	29.00 [14.00, 61.00]
Total hospitalization cost (2021 CNY), median [IQR]	63575.49 [22144.50, 115280.17]
Cost per day (2021 CNY), median [IQR]	1747.00 [1250.73, 2603.39]
Insurance type (%)	
UEBMI	3632 (14.3)
URRBMI	15012 (59.3)
Self-pay	5359 (21.2)
Other	1328 (5.2)
Surgical grade (%)	
No surgery	19981 (78.9)
Grade 1	676 (2.7)
Grade 2	356 (1.4)
Grade 3	687 (2.7)
Grade 4	3631 (14.3)

Values are presented as mean (SD), median [IQR], or number (%), as appropriate. Costs were adjusted for inflation using the consumer price index (CPI) and expressed in 2021 Chinese Yuan (CNY).

### Comparison before and after performance reform

A total of 10,653 patients were admitted in the pre-reform period (2016–2017), and 10,482 were admitted in the post-reform period (2019–2020) (**Supplementary Table S1**). Patient age and sex distribution were broadly comparable between the two periods, although standardized mean differences suggested moderate imbalance in admission frequency and surgical characteristics. Median length of stay decreased from 30 days (IQR, 13–66) before the reform to 27 days (IQR, 14–55) after the reform. In contrast, median total hospitalization costs increased from 54,683.92 to 72,732.25, and median daily costs increased from 1,509.91 to 2,203.69. The proportion of patients undergoing surgery, particularly high-grade procedures, was higher in the post-reform period.

### Interrupted time series analysis of inpatient outcomes

**Figure 1:**
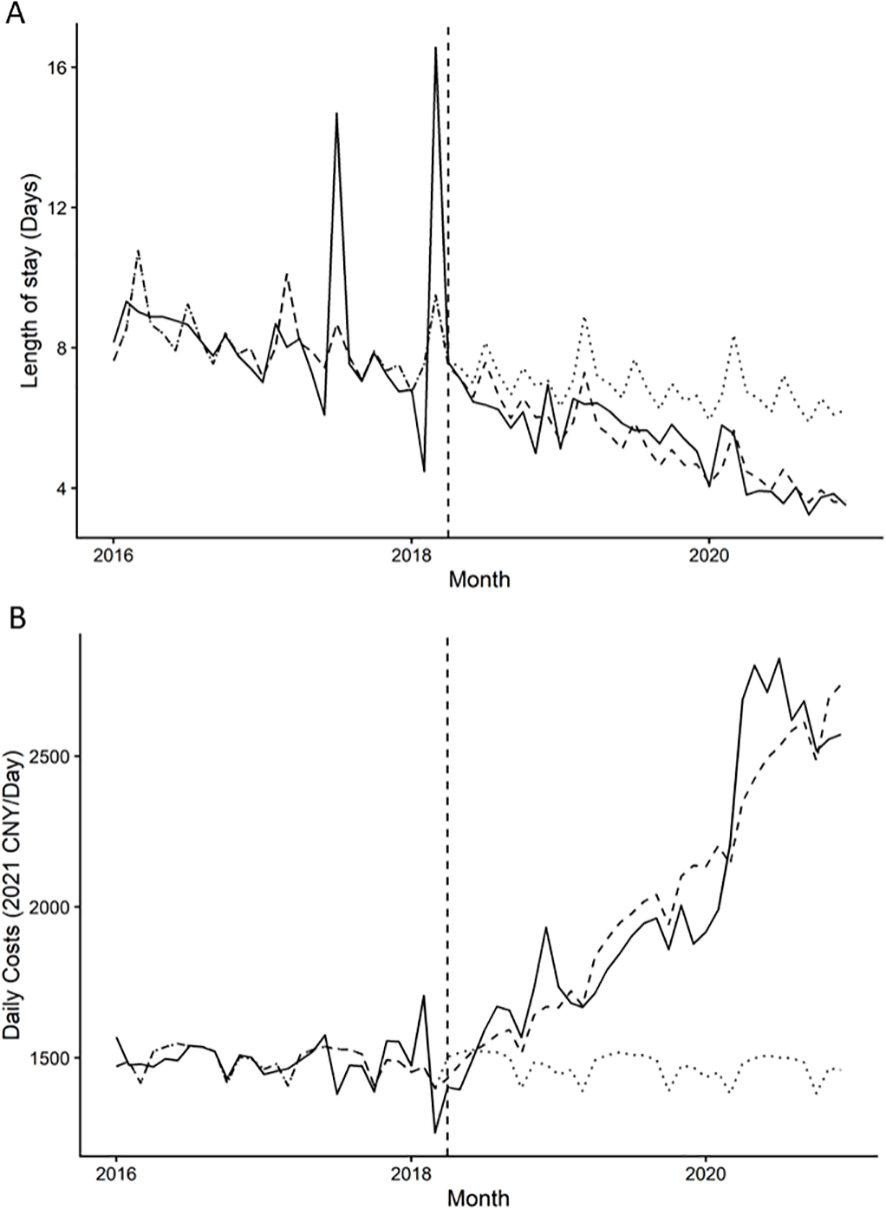
Interrupted time series of inpatient outcomes before and after the 2018 hospital performance reform. Points represent monthly geometric means. Graph **(A)** shows length of stay (days), and Graph **(B)** shows daily hospitalization costs (2021 CNY per day), from 2016 to 2020. Solid lines indicate fitted segmented regression trends, and the vertical dashed line marks the implementation of the reform in April 2018.

**Figure 1**, **Table 2** present the results of the ITSA assessing monthly trends in inpatient outcomes associated with the April 2018 performance reform. For length of stay, no statistically significant immediate level change was observed at the time of reform implementation (−2.53%, p = 0.77). However, a significant change in post-reform trend was detected, with length of stay decreasing by an estimated 1.59% per month relative to the pre-reform trend (p < 0.001) (**Figure 1A**). For daily hospitalization costs, no significant immediate level change was observed (−4.61%, p = 0.363). In contrast, daily costs exhibited a significant upward post-reform trend, increasing by approximately 2.14% per month compared with the pre-reform period (p < 0.001) (**Figure 1B**). Results were directionally consistent when excluding 2020 and when restricting to index admissions (**Supplementary Figures S2**, **S3**), supporting robustness to potential COVID-19 disruptions and repeated-admission effects.

**Table 2:** Interrupted time series analysis of the association between hospital performance reform and inpatient outcomes among patients with lung cancer.

Outcome	Parameter	Coefficient (β)	% Change	*P*-value
Log (LOS) (geometric mean, days)	Level change (post)	-0.026	-2.53	0.77
	Slope change (per month, time after)	-0.016	-1.59	<0.001
Log (Daily costs) (geometric mean, 2021 CNY/day)	Level change (post)	-0.047	-4.61	0.363
	Slope change (per month, time after)	0.021	2.14	<0.001

Models were estimated using segmented regression with April 2018 as the intervention breakpoint. Monthly outcomes were log-transformed. Models adjusted for seasonality using month indicators. Newey–West standard errors were applied to account for autocorrelation.

### Patient-level regression analyses

As secondary analyses, patient-level regression models were conducted to assess consistency with the ITSA findings (**Table 3**). Compared with the pre-reform period, patients admitted during 2019–2020 experienced significantly shorter length of stay (β = −0.174, 95% CI −0.194 to −0.154; p < 0.001) and higher daily hospitalization costs (β = 0.277, 95% CI 0.263 to 0.291; p < 0.001). In sensitivity analyses restricted to admissions in 2019 only, excluding 2020, the associations remained statistically significant, although effect sizes were attenuated (**Table 3**). Analyses of total hospitalization costs are presented in the **Supplementary Materials** (**Supplementary Table S2**), where the post-reform period was associated with higher total costs (β = 0.103, 95% CI 0.083 to 0.124; p < 0.001).

**Table 3:** Association between hospital performance reform and inpatient outcomes among patients with lung cancer (Patient-Level, 2016–2020, Log-Transformed).

Variables	Length of stay		Cost per inpatient day	
	β (95% CI)	*P*-value	β (95% CI)	*P*-value
Main analysis				
Reform				
2016–2017	Ref		Ref	
2019-2020	-0.174 (-0.194, -0.154)	<0.001	0.277 (0.263, 0.291)	<0.001
Sensitivity analysis				
Reform				
2016–2017	Ref		Ref	
2019	-0.059 (-0.083, -0.037)	<0.001	0.167 (0.150, 0.183)	<0.001

## Discussion

This study utilized real-world inpatient administrative data from a tertiary cancer hospital in China to assess the burden of lung cancer hospitalizations and examine changes associated with a hospital-wide performance reform implemented in April 2018. The reform adjusted internal performance evaluation and income distribution, placing greater emphasis on advanced surgical procedures and clinical workload. We observed substantial inpatient burden, with prolonged LOS and high costs. Using ITSA, we found no evidence of an immediate level change at the intervention point, but observed significant post-reform slope changes: LOS declined more rapidly over time, while daily costs increased at a faster rate. Compared with the pre-reform period, the post-reform period was associated with an estimated 16.0% reduction in LOS, alongside a 31.9% rise in daily costs. These patterns suggest potential improvements in time-related efficiency accompanied by increased resource utilization intensity in specialized oncology care.

The reduction in LOS may reflect enhanced operational efficiency, such as improved bed turnover and care coordination, consistent with the reform’s incentives (16). The presence of slope (trend) changes rather than an abrupt level shift is plausible because hospital-wide performance reforms often take time to be implemented and absorbed into clinical workflows. Similar shortening of hospital stays has been reported in evaluations of other Chinese hospital reforms, including diagnosis-related group (DRG)-based payment pilots and zero-markup drug policies, where efficiency gains often coincided with structural shifts in cost composition (17–20).

In contrast, the marked increase in daily costs likely indicates greater care intensity per hospital day. The reform’s weighting toward advanced procedures was accompanied by higher proportions of surgical and high-grade interventions, which may have contributed to increased use of complex diagnostics, consumables, and therapeutic approaches (21). These associations may also reflect broader advances in lung cancer management during the study period, including growing adoption of more resource-intensive treatments, as well as contemporaneous system-level reforms (e.g., national salary reforms and payment changes) that could not be fully disentangled here (22, 23). In addition, several national health system policies and secular trends during the study period (e.g., payment reforms and procurement policies) may have concurrently influenced LOS and cost intensity; therefore, our findings should be interpreted as associations rather than causal effects attributable solely to the hospital reform.

Together, these findings illustrate a common trade-off in specialized settings: gains in throughput efficiency may coexist with escalated resource intensity. Relying on single indicators risks incomplete interpretation—focusing solely on LOS might overstate efficiency improvements, while emphasizing costs alone could obscure organizational gains (7). Our multi-indicator approach provides a more nuanced perspective and highlights the value of complementary metrics when assessing performance reforms in oncology (24).

From a policy standpoint, these results are particularly relevant for tertiary cancer hospitals, where prolonged baseline LOS and specialized care pathways are common (14). Performance-oriented reforms can encourage complex service delivery but may contribute to cost escalation if not balanced with measures to control intensity, such as strengthened clinical pathways or oversight of high-cost items (7, 25, 26). Whether increased care intensity translates into better patient outcomes or raises concerns about potential overuse warrants further investigation with clinical and quality data. Because we did not have patient outcome measures (e.g., complications, readmissions, survival, or patient-reported outcomes), we cannot determine whether higher daily costs reflect value-improving clinical advances or unintended increases in service intensity. Sensitivity analyses excluding 2020 and restricting to index admissions produced directionally consistent results, supporting robustness to potential COVID-19 disruptions and repeated-admission effects.

Several limitations should be noted. First, as a single-center study in a specialized oncology hospital, findings may not generalize to general hospitals or lower-tier facilities, though they offer targeted insights for similar cancer centers. Second, reliance on administrative data limited adjustment for clinical details (e.g., tumor stage, comorbidities, or specific therapies), potentially leading to residual confounding. Third, the observational design precludes causal inference, and unmeasured contemporaneous factors may have influenced observed patterns. Finally, the analysis focused on inpatient outcomes and did not capture outpatient or post-discharge utilization. Despite these limitations, this study provides disease-specific, real-world evidence on lung cancer inpatient burden and reform-associated changes. By integrating efficiency- and cost-related indicators, it contributes to understanding how internal performance management reforms may reshape resource utilization in oncology, informing efforts to achieve sustainable efficiency, quality, and affordability in China’s specialized hospital sector.

## Conclusion

In this real-world study of patients hospitalized with lung cancer, the hospital performance reform implemented in April 2018 was associated with gradual changes in inpatient utilization patterns. These findings suggest a potential efficiency–intensity trade-off in specialized oncology care and underscore the importance of using multiple, complementary indicators—rather than single metrics—when evaluating hospital management reforms.

## Data Availability

The original contributions presented in the study are included in the article/**Supplementary Material**, further inquiries can be directed to the corresponding author(s).
