# Supplementary material for: Inpatient burden of lung cancer and changes after a hospital performance reform: a real-world study
Source: Front Oncol. 2026 Jan 26;15:1771441. doi: 10.3389/fonc.2025.1771441 (PMC12883420; doi:10.3389/fonc.2025.1771441)
Supplement: Supplementary file 1 [file Supplementaryfile1.docx]

**Supplementary Materials**

**Table S1** Comparison of patient characteristics and inpatient burden before and after the hospital performance reform

| **Variables** | **Pre-reform (*N*=10653)** | **Post-reform (*N*=10482)** |
| --- | --- | --- |
| Age, mean (SD) | 60.45 (10.43) | 61.39 (10.46) |
| Sex, % male | 6975 (65.5) | 6651 (63.5) |
| **Hospitalization characteristics** |  |  |
| Number of admissions, median [IQR] | 2.00 [1.00, 6.00] | 2.00 [1.00, 7.00] |
| Total length of stay (days), median [IQR] | 30.00 [13.00, 66.00] | 27.00 [14.00, 55.00] |
| Total hospitalization cost (2021 CNY), median [IQR] | 54683.92 [18590.23, 105097.37] | 72732.25 [27813.11, 127003.78] |
| Cost per day (2021 CNY), median [IQR] | 1509.91 [1139.49, 2091.05] | 2203.69 [1516.62, 3253.29] |
| **Insurance type (%)** |  |  |
| UEBMI | 942 (8.8) | 2016 (19.2) |
| URRBMI | 6031 (56.6) | 6457 (61.6) |
| Self-pay | 3153 (29.6) | 1474 (14.1) |
| Other | 527 (4.9) | 535 (5.1) |
| **Surgical grade (%)** |  |  |
| No surgery | 9047 (84.9) | 7452 (71.1) |
| Grade 1 | 87 (0.8) | 553 (5.3) |
| Grade 2 | 170 (1.6) | 152 (1.5) |
| Grade 3 | 290 (2.7) | 284 (2.7) |
| Grade 4 | 1059 (9.9) | 2041 (19.5) |

Values are presented as mean (SD), median [IQR], or number (%), as appropriate. Costs were adjusted for inflation using the consumer price index (CPI) and expressed in 2021 Chinese Yuan (CNY).

**Table S2** Multivariable Linear Regression Analysis of Total Hospitalization Costs Among Patients with Lung Cancer (Log-Transformed)

| Variables | Total costs | |
| --- | --- | --- |
|  | β (95% CI) | *P*-value |
| Reform |  |  |
| 2016–2017 | Ref |  |
| 2019-2020 | 0.103 (0.083, 0.124) | <0.001 |
| Age (per 1-year increase) | 0.002 (<0.001, 0.002) | 0.003 |
| Sex |  |  |
| Male | Ref |  |
| Female | -0.045 (-0.066, -0.024) | <0.001 |
| Insurance type |  |  |
| UEBMI | Ref |  |
| URRBMI | -0.067 (-0.097, -0.037) | <0.001 |
| Self-pay | -0.080 (-0.115, -0.045) | <0.001 |
| Other | -0.042 (-0.094, 0.009) | 0.108 |
| Surgical grade |  |  |
| No surgery | Ref |  |
| Low-grade surgery (1–2) | 0.428 (0.379, 0.476) | <0.001 |
| High-grade surgery (3–4) | 1.012 (0.985, 1.039) | <0.001 |
| Log of number of admissions | 0.814 (0.803, 0.824) | <0.001 |


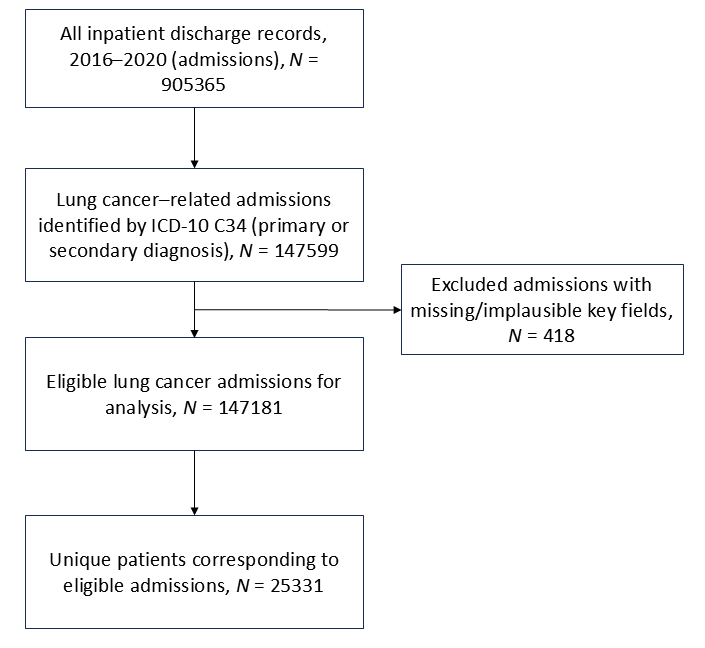


**Figure S1** Flow Diagram of Cohort Identification and Analytic Sample Selection


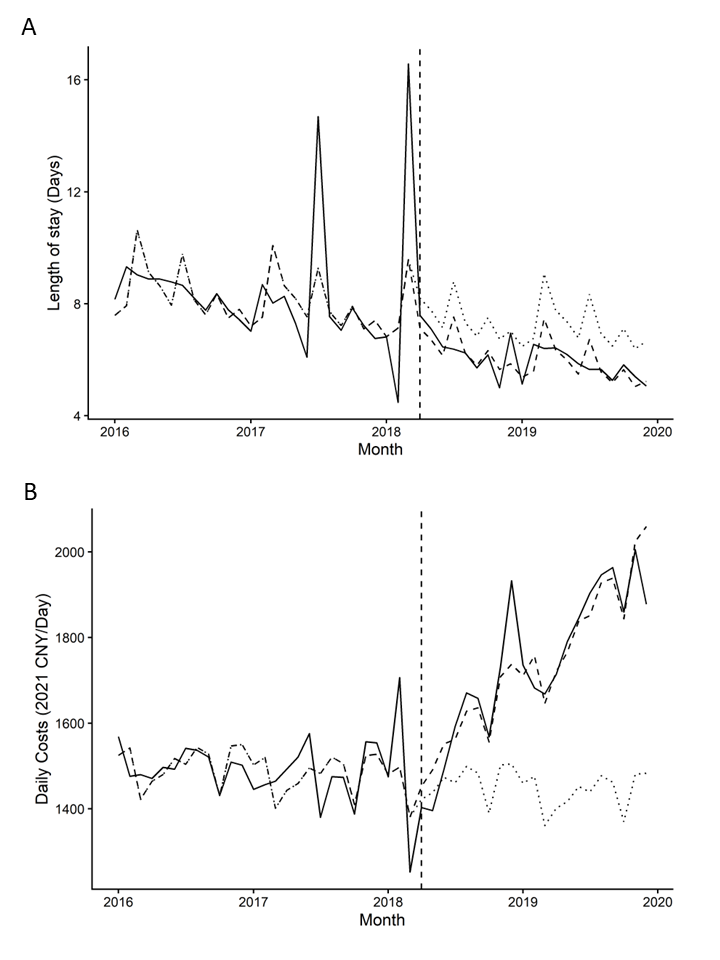


**Figure S2** Interrupted Time Series Analysis Excluding the Year 2020

Note: Sensitivity analysis conducted to assess robustness to potential COVID-19–related disruptions. Points represent monthly geometric means. Solid lines indicate fitted segmented regression trends. The vertical dashed line denotes the reform implementation in April 2018.


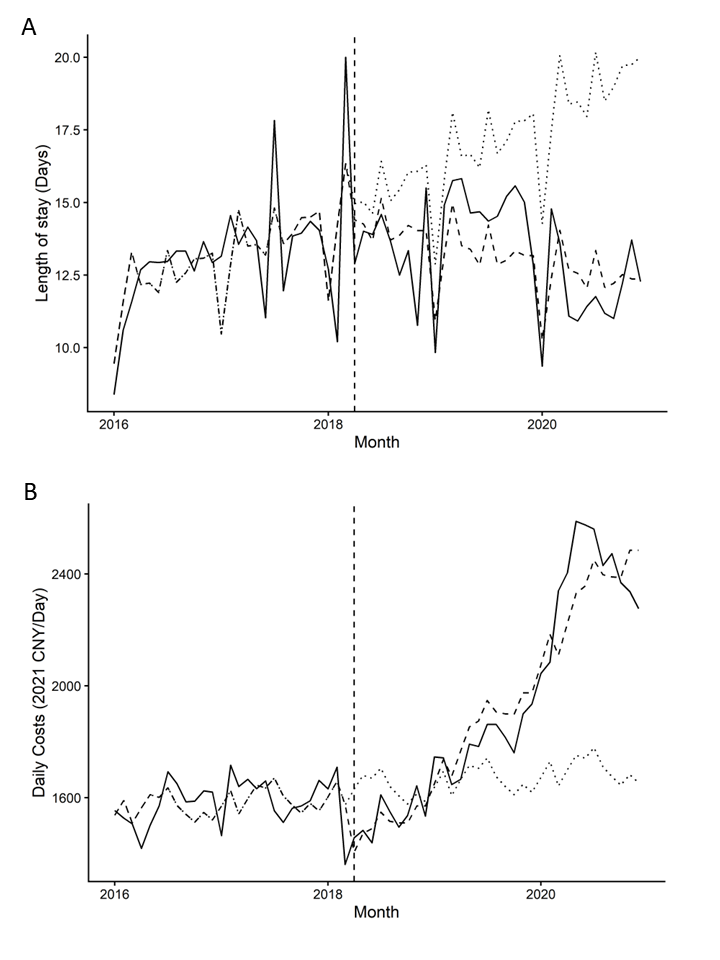


**Figure S3** Interrupted Time Series Analysis Restricted to Index Admissions (First Hospitalization per Patient)

Note: Points represent monthly geometric means. Solid lines indicate fitted segmented regression trends. The vertical dashed line denotes the reform implementation in April 2018.
